# Convolutional neural networks for classifying healthy individuals practicing or not practicing meditation according to the EEG data

**DOI:** 10.18699/VJGB-23-98

**Published:** 2023-12

**Authors:** X. Fu, S.S. Tamozhnikov, A.E. Saprygin, N.A. Istomina, D.I. Klemeshova, A.N. Savostyanov

**Affiliations:** Novosibirsk State University, Novosibirsk, Russia; Scientific Research Institute of Neurosciences and Medicine, Novosibirsk, Russia; Scientific Research Institute of Neurosciences and Medicine, Novosibirsk, Russia Institute of Cytology and Genetics of the Siberian Branch of the Russian Academy of Sciences, Novosibirsk, Russia; Novosibirsk State University, Novosibirsk, Russia; Institute of Cytology and Genetics of the Siberian Branch of the Russian Academy of Sciences, Novosibirsk, Russia; Novosibirsk State University, Novosibirsk, Russia Scientific Research Institute of Neurosciences and Medicine, Novosibirsk, Russia Institute of Cytology and Genetics of the Siberian Branch of the Russian Academy of Sciences, Novosibirsk, Russia

**Keywords:** convolutional neural networks, EEG, event-related brain potentials, meditation, stop-signal paradigm, сверточные нейронные сети, ЭЭГ, вызванные потенциалы мозга, медитация, парадигма стоп-сигнал

## Abstract

The development of objective methods for assessing stress levels is an important task of applied neuroscience. Analysis of EEG recorded as part of a behavioral self-control program can serve as the basis for the development of test methods that allow classifying people by stress level. It is well known that participation in meditation practices leads to the development of skills of voluntary self-control over the individual’s mental state due to an increased concentration of attention to themselves. As a consequence of meditation practices, participants can reduce overall anxiety and stress levels. The aim of our study was to develop, train and test a convolutional neural network capable of classifying individuals into groups of practitioners and non-practitioners of meditation by analysis of eventrelated brain potentials recorded during stop-signal paradigm. Four non-deep convolutional network architectures were developed, trained and tested on samples of 100 people (51 meditators and 49 non-meditators). Subsequently, all structures were additionally tested on an independent sample of 25 people. It was found that a structure using a one-dimensional convolutional layer combining the layer and a two-layer fully connected network showed the best performance in simulation tests. However, this model was often subject to overfitting due to the limitation of the display size of the data set. The phenomenon of overfitting was mitigated by changing the structure and scale of the model, initialization network parameters, regularization, random deactivation (dropout) and hyperparameters of cross-validation screening. The resulting model showed 82 % accuracy in classifying people into subgroups. The use of such models can be expected to be effective in assessing stress levels and inclination to anxiety and depression disorders in other groups of subjects.

## Abstract (ru)

В настоящее время разработка объективных методик для оценки уровня стресса является чрез- вычайно актуальной задачей прикладной нейронауки. Анализ электроэнцефалограммы (ЭЭГ), записанной в условиях выполнения заданий на самоконтроль поведения, может служить основой для разработки тестовых методик, позволяющих классифицировать людей по уровню стресса. Хорошо известно, что одним из следствий медитационной практики является выработка у участников навыков произвольного контроля над собственным ментальным состоянием за счет повышенной концентрации внимания на самом себе. На фоне медитационной практики часто происходит снижение общего уровня тревожности и стресса. Целью нашего исследования было разработать, обучить и протестировать сверточную нейронную сеть, способную классифицировать людей на группы участвующих или не участвующих в медитационной практике на основе анализа вызванных потенциа- лов головного мозга, записанных при выполнении заданий парадигмы стоп-сигнал. Были разработаны четыре архитектуры неглубоких сверточных сетей, которые были обучены и протестированы на выборке из 100 че- ловек (51 медитатор и 49 не-медитатор). В дальнейшем все структуры были дополнительно протестированы на независимой выборке в 25 человек. Установлено, что структура, использующая одномерный сверточный слой, который объединяет слой и двуслойную полностью подключенную сеть, показала наилучшие результаты работы в имитационных тестах. Однако эта модель была часто подвержена переобучению из-за ограничения размера отображения набора данных. Явление переобучения было смягчено при помощи изменения структу- ры и масштаба модели, параметров сети инициализации, регуляризации, случайной деактивации (dropout) и гиперпараметров скрининга перекрестной проверки. В итоге нами получена модель, которая показала 82 % точность в классификации людей на подгруппы. Можно ожидать, что использование таких моделей окажется эффективным методом для оценки уровня стресса и предрасположенности к тревожным и депрессивным рас- стройствам в других группах испытуемых.

## Introduction

Stress is one of the most common problems in modern society, and the search for effective methods to assess stress levels is important for early detection of the risk of mental and psychosomatic disorders (Kuh et al., 2003; Kuznetsova et al., 2016). Most psychological methods of assessing stress levels are based on the use of questionnaires, in which the respondent answers questions about their subjective mental condition. The weak point of this approach is the high probability of incorrect self-assessments, arising either from a person’s unwillingness to report their problems, or as a result of a low ability to recognize changes in their own condition (Iwata, Higuchi, 2000; McCrae et al., 2000). A possible solution to this problem is to develop objective approaches to the diagnosis of mental traits or conditions based on the analysis of brain signals, such as fMRI or EEG.

Meditation is a system of special mental practices aimed at establishing voluntary self-control over one’s mental state. Although meditation initially appears as part of religious practices, especially common in oriental religions, at present this phenomenon is a popular topic of interest among scientific researchers. Meditation is considered as a basis for the creation of non-invasive, non-drug techniques that reduce the risk of a wide range of mental or psychosomatic diseases. A number of studies have shown that meditation has many positive effects on mental health, including a general reduction in stress and the level of propensity to depression (Chiesa et al., 2011; Saeed et al., 2019). The analysis of the EEG recorded during recognition of emotional stimuli revealed significant effects of meditation on the state of the human brain (Aftanas, Golosheykin, 2005; Atchley et al., 2016; Savostyanov et al., 2020). Therefore, the comparison of EEG in practitioners and non-practitioners of meditation can be considered as an experimental model that allows the development of methods for assessing stress levels

Stop-signal paradigm (SSP) is an experimental method for evaluating an individual’s ability for voluntary self-control of their own movements in a changing external environment (Logan, Cowan, 1984; Band et al., 2003). The SSP allows us to assess the balance of two processes – activation and inhibition of behavior under conditions of insufficient time for making a decision. A number of studies have shown that SSP is an effective method for diagnosing the level of personal anxiety and propensity to depression (Hsieh et al., 2021; Zelenskih et al., 2022). It can be assumed that the dynamics of brain activity during SSP will serve as a marker distinguishing practitioners and non-practitioners of meditation from each other.

Artificial neural network is a developing technology based on machine learning, which is widely used in various fields. Compared to other traditional methods of machine classification, such as linear discriminant analysis and the k-nearest neighbor algorithm, artificial neural networks provide more accurate results of classifying individuals according to their behavioral and neurophysiological characteristics (Khosla et al., 2020). Therefore, in comparison with the support vector machine, an artificial neural network is better suited for the tasks of multiple classification, providing convenience for further research, as well as more efficient fitting of complex nonlinear relationships.

The purpose of our research is to develop, train and test an artificial neural network that allows, based on the analysis of event-related brain potentials in the stop-signal paradigm, to classify individuals according to the criterion of whether they practice meditation. We assume that afterwards the neural network created in this way will be able to assess individual level of stress and propensity to anxiety-depressive disorders.

## Materials and methods

Participants. A group of people practicing samadhi meditation (also called “mindfulness meditation”) was examined in July–August 2018 on the premises of the Baikal Retreat Center (http://www.geshe.ru/). The experimental group included 51 healthy, right-handed participants from 25 to 66 years old (32 men; average age = 41.0, SD = 8.3), practicing meditation for a period of 5 to 15 years. The control group was examined in October–November 2019 on the premises of the medical college of the village of Khandyga, Tomponsky district of the Republic of Sakha (Yakutia). The control group included 49 healthy, right-handed participants from 22 to 58 years old (22 men; average age = 38.0, SD = 8.3) who had never participated in meditation or yoga practices

The protocol of the study was approved by the local Ethics Committee of the Research Institute of Neurosciences and Medicine in accordance with the Helsinki Declaration of Biomedical Examinations. All the participants signed informed consent to participate in the surveys.

Experimental procedure. The experiment was organized on the basis of the stop-signal paradigm proposed in 1984 (Logan, Cowan, 1984) and modified by A.N. Savostyanov and co-authors (Savostyanov et al., 2009). The experiment was organized in the form of the computer interactive game “Hunt”. One of two images appeared on the computer screen: a deer, or a tank. The participant had to press the keyboard button corresponding to the picture. The response time was limited to 0.75 seconds. If the participant pressed the button correctly and faster than 0.75 seconds, their game score increased. If the participant pressed the buttons incorrectly or reached a time out, then their game score decreased.

In total, 135 stimuli were presented to each participant. In 35 cases, after the onset of the target signal, a stop-signal was presented (a red square with the inscription “Stop”), which meant that the participant had to interrupt the movement that had already begun. If the participant did not press the button after the stop-signal, their score did not change. If the participant pressed the button after the stop-signal, their score decreased. The order of activation and stopping trials was randomized. The sequence of “deer” and “tank” stimuli was also randomized. The interval between the end of the previous task and the start of a new task varied from 3 to 7 seconds. The total duration of the experiment was approximately 12 minutes.

Preprocessing of experimental data. EEG rejection of artifacts was done by the ICA method (Delorme, Makeig, 2004). The initial EEG signal was filtered at 1–40 Hz and referenced to average of all channels. The data was epoched relative to the onset trigger of the target stimulus (deer or tank) at a time interval from –1 to +3 seconds. The baseline EEG level was set in the range from –1000 to –250 ms. In total, 80 to 90 EEG epochs were obtained for each participant, after exclusion of all the trials containing the stop-signal or artifacts. After excluding artifacts, event-related potentials (ERPs) were calculated separately for each EEG channel, averaged over all trials and all participants

The ERP calculation was conducted in the ERPLAB toolbox for MATLAB. Amplitude-time ERP graphs were made for each EEG channel. Then a visual preview of the ERP graph for the C3 channel was performed. In this lead, the ERP motor peaks stand out the most. In particular, two peaks were selected for this lead – an early premotor peak, the amplitude of which precedes pressing the button (the so-called readiness potential) and a late motor peak, the amplitude of which reaches a maximum when the button is pressed. From viewing this visual, the time limits of both the early and late peak were established. After that, the amplitude in each of these time windows was calculated separately for each person and each EEG channel, but averaged over all trials of the activation condition of the task for each participant. The calculation of the averaged amplitude was made using the ERPLAB toolbox (https://erpinfo.org/erplab). The amplitude values were surveyed to the baseline level for each participant separately. The obtained values were used as training and test data for artificial neural networks.

**Fig. 1.**
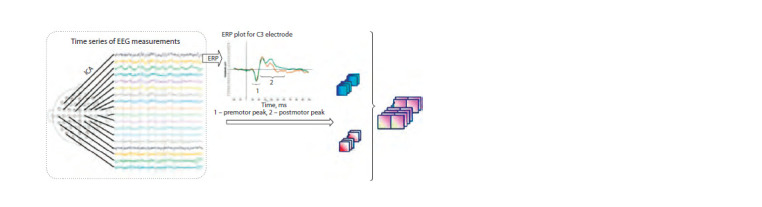
The scheme of obtaining input data for the neural network.

EEG data acquisition. The general structure of the input data is shown in Figure 1. For each participant, EEG was analyzed for 64 channels located at different points of the head surface. According to the international scheme of 10–20 %, the name of the electrode reflects its spatial position. The initial EEG signal for each channel is presented as a continuous series of measurements of the potential difference between the surface electrode and the referent with a time resolution of 1,000 measurements per second.

ERP extraction. When calculating the ERP (event-related potential) amplitude, the researcher selects several time windows, in each of which all amplitude values are summed over all time points and averaged over all tests. The amplitude values in different windows reflect the temporal dynamics of the neurophysiological process. We selected two time windows (250–350 and 550–900 ms after the target signal), which reflect, respectively, the physiological processes associated with the preparation and execution of the movement. A numerical value of the ERP amplitude was obtained for each participant separately for each time window and for each EEG channel. Since ERP in different parts of the head can deviate from the zero value of the potential both up (positive peak) and down (negative peak), then the numerical values of the amplitude can be both positive and negative. Thus, our data takes into account both spatial (the name of the channel, i. e. its position on the head) and temporal (the first or second ERP window) characteristics of the brain response to the task in the stop-signal paradigm, as well as the electrical direction of the reaction (positive or negative peak amplitude values).

For each examined individual, the data dimension was 2×64 values. Since 50 participants were included in each group of people, the data size for each of our samples is approximately 50×2×64, and the total size of the data set is 100×2×64.

Designing the structure and framework of a neural network

Since the input set of ERP data is small, a non-deep neural network was designed to predict whether an individual participated in long-term meditations or not. However, the initial EEG recording also has time series characteristics, so a convolutional neural network was additionally used for its analysis as a deep neural network for training and prediction. The main components of the convolutional neural network include convolutional layers, pooling layers, and fully connected layers.

In our case, the input layer of the convolutional network receives EEG data transformed into a two-dimensional matrix with a sample size of 2×64, where each row represents an individual ERP peak and each column represents an EEG recording channel. The hidden layer of the convolutional neural network includes three common architectures: a convolutional layer, a pooling layer, and a fully connected layer. We used the Conv1d() tool in PyTorch as the convolutional kernel, which prevented overfitting caused by using more complex convolutional kernels with more parameters (https://pytorch.org/docs/stable/generated/torch.nn.Conv1d. html#torch.nn.Conv1d, 21.02.2023).

The parameters of the convolutional layer include the kernel size, stride size, and padding, which collectively determine the size of the output feature map of the convolutional layer and are hyperparameters of the convolutional neural network. Due to the characteristics of EEG data, there are both spatial and temporal relationships, so we developed two schemes. The first scheme involves using a total of two one-dimensional convolutions. One extracts spatial features, which represent connections between ERP peaks in different electrode channels, and the other extracts temporal features. In this scheme, the PyTorch Conv1d() function wrapper was used to complete the corresponding function. The second approach involves applying only one one-dimensional convolution, but this convolution can extract both temporal and spatial features, for which the PyTorch Conv1d() function wrapper was also chosen.

The convolutional layers contain activation functions that help represent complex objects. In our study, three activation functions were used: sigmoid(), relu(), and softmax() from PyTorch (https://pytorch.org/docs/stable/generated/torch. nn.BCELoss.html, 15.04.2023). After extracting objects in the convolutional layer, the output feature map was passed to the pooling layer for object selection and information filtering. The pooling layer selects the pooling region in the same way as the kernel scanning stage of the convolutional layer, which is controlled by the pooling size, stride size, and padding. The convolutional and pooling layers in the convolutional neural network can extract features from the input data. The role of the fully connected layer is to nonlinearly combine the extracted features to obtain output data. In our case, two fully connected layers were created to prevent overfitting due to the small size of the dataset, for which the Linear() tool in PyTorch was applied. A fully connected layer is typically located before the output layer in a convolutional neural network. We used different loss and activation functions during training based on these two scenarios to improve the accuracy and performance of the model.

**Fig. 2.**
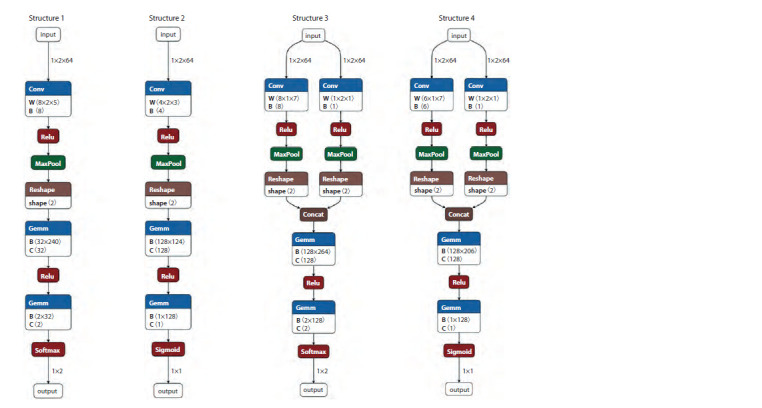
Flowcharts of four models (structures) for the neural network architecture.

According to the above-described scheme, four network structures were designed and used for classifying surveyed individuals (Fig. 2). The only difference between these four architectures lies in the number of convolutional layers and the number of output neurons at the end.

In the first structure, a convolutional layer is used to extract both temporal and spatial objects. Then, two fully connected layers are used, and two values are output after normalization using the softmax activation function. Cross-entropy is used as the loss function, and Adam is used as the gradient descent algorithm.

The second structure also uses a convolutional layer to extract both temporal and spatial objects. Then, two fully connected layers are used, and the value is output after activation with the sigmoid function. Binary cross-entropy is used as the loss function, and Adam is used as the gradient descent algorithm.

The third structure uses two types of convolutions to extract spatial and temporal characteristics of the data, respectively. Then, two fully connected layers are used, and two values are output after normalization using the softmax activation function. Cross-entropy is used as the loss function, and Adam is used as the gradient descent algorithm.

Finally, the fourth structure uses two types of convolutions to extract spatial and temporal characteristics of the data, respectively. Then, two fully connected layers are used, and the value is output after activation with the sigmoid function. Binary cross-entropy is used as the loss function, and Adam is used as the gradient descent algorithm.

Optimal hyperparameters were found for each structure and are described in the model evaluation section.

Neural network training

The process of training an artificial neural network can be divided into four stages: initialization, forward propagation, backward propagation, and weight update.

During initialization, we assigned random initial values to each parameter (weights and biases) of the neural network to break symmetry and allow each neuron to have a different gradient and learn different functions. Later, during hyperparameter search, we determined the optimal initialization function for each architecture. During forward propagation, the training data (input and output) were fed into the neural network, and the activation value of each neuron was calculated sequentially from the input layer to the hidden layer, and then to the output layer according to the structure of the neural network. The activation values were obtained from the linear combination of the input data and weights plus bias, followed by a non-linear function such as sigmoid or ReLU. The goal of forward propagation was to obtain the predicted result of the neural network and compare it with the true result. The goal of backward propagation was to obtain the gradient of each parameter, which can be used to update the parameters. In our case, we used cross-entropy loss function and binary cross-entropy loss function for this purpose (https://pytorch. org/docs/stable/generated/torch.nn.CrossEntropyLoss.html, 20.03.2023). The cross-entropy loss function was used to measure the distance between the probability distribution predicted by the model and the true probability distribution. Using this, we evaluated the performance of the model and selected the optimal model and parameter by comparing the loss values of different models or different parameters.

Each parameter is updated with a certain learning rate (step size) according to its gradient, so that the loss function decreases. The goal of weight update is to optimize the parameters of the neural network so that it can better fit the training data. For this task, we applied the Adam optimization method. Adam is an algorithm for stochastic gradient descent with adaptive momentum, which was proposed at the ICLR conference in 2015 and has become one of the most popular and effective optimizers in deep learning. Adam combines two classical optimization algorithms, Adagrad and RMSProp, which are capable of handling sparse gradients and non-stationary objective functions, and uses the idea of momentum to accelerate convergence. Adam is equivalent to having a separate learning rate for each parameter, and this learning rate is adaptively adjusted according to the change in gradient. Specifically, when the gradient is large, the estimate of the second moment increases, which reduces the learning rate. When the gradient is small or sparse, the estimate of the first moment increases, which increases the learning rate. This effectively avoids oscillations caused by a too large learning rate, or increased complexity of convergence caused by a too small learning rate, or even getting trapped in a local minimum or saddle point.

To reduce overfitting and better train the model, we used batch normalization. Batch normalization is an approach that solves the problem of vanishing gradients by improving the smoothing of losses, speeding up network convergence, and increasing accuracy (Loffe, Szegedy, 2015). This method normalizes the data in mini-batches so that the mean value is 0 and the standard deviation is 1. At the same time, two trainable parameters, scale and shift, are introduced so that the model can learn its corresponding distribution during backward propagation. To implement this function, we used the BatchNorm1d() tool from PyTorch.

Overfitting is a common problem in the process of training an artificial neural network, where the model performs well on the training set but poorly on the test set or new data, indicating poor generalization. In our case, the problem was in overfitting due to a small dataset. To solve this problem, we applied initialization, L2 regularization, and dropout, as well as cross-validation to evaluate the model and select hyperparameters that best train the model, reducing overfitting to some extent. We used L2 regularization (weight decay), which involves adding a penalty term to the loss function proportional to the sum of squares of the model’s parameters. L2 regularization can cause the model’s parameters to tend towards smaller values, thereby reducing the model’s sensitivity to noise or outliers. Random deactivation (dropout) means the random zeroing of certain neurons or connection layers with a certain probability during training, which reduces the number of model parameters, thereby increasing the reliability and generalization ability of the model.

Cross-validation is the reuse of data, splitting the resulting dataset, combination into various training and test sets, a training set for training the model and a test set for evaluating the quality of model prediction. We used the K-fold multiplication method as a cross-validation method to reduce overfitting.

Evaluation of model performance on training data

In accordance with the characteristics of the EEG data sample and the indicators of the benchmark classification model, we used the metrics “F1-score”, “AUC” (area under curve), and “accuracy” as evaluation indicators for the model (https://keras.io/api/models/model_training_apis). The higher these indicators, the better the model’s performance. F1-score and AUC are comprehensive evaluation indicators for classification models, but they have different inaccuracies. AUC is less affected by the ratio of positive and negative samples in the dataset. For the purposes of this development, it became clear that predicting a person with a high level of stress as a person with a low level of stress would mean fundamentally incorrect results. Therefore, we chose F1-score as the most prioritized indicator for evaluating the model’s effectiveness. We evaluated the model’s hyperparameters using five-fold cross-validation to select the most suitable hyperparameters to prevent overfitting and improve model performance.

**Fig. 3.**
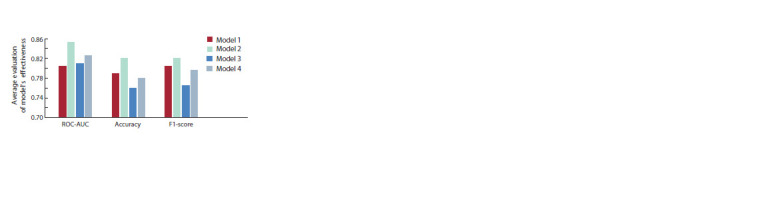
Results of testing four different neural network models on the training sample

The results of evaluating the model on the training dataset are presented in Figure 3. Looking at each of the selected indicators, we can see that model 2 showed the most effective classification. Its effectiveness exceeded 80 % for all selected indicators. Models 1 and 4 also show good classification results, while model 3 performs the worst. Therefore, we assume that the output of one neuron surpasses the use of two neurons in the EEG binary classification task. Binary cross-entropy loss is obviously more suitable for our classification task based on the available dataset. When evaluating the model’s effectiveness, the number of samples was 100, with 51 individuals practicing meditation (low stress level) and 49 individuals not practicing meditation. The number of samples is balanced, so it does not significantly affect the training and performance of the model. Moreover, for data with only two ERP peaks in 64 electrode channels, one convolution extracting both temporal and spatial characteristics worked better than two convolutions extracting temporal and spatial characteristics separately.

Evaluation of model performance on independent data.

To evaluate the performance of the model on independent data, we prepared EEG data obtained from 25 individuals who were not included in the training set. Out of these 25 individuals, 12 practiced meditation, while 13 did not. The equipment, experimental design, and preprocessing of the EEG data were the same as in the training set. In this part of the study, all previously trained models were tested on new data that was not included in the training set. Accuracy, reliability, responsiveness, F1-score, ROC-AUC, specificity, and sensitivity were used as performance indicators for evaluating the models. Despite using parameter initialization functions, the weights were still randomly initialized within a certain range. Therefore, we adjusted the initial value of the random number to ensure the stability of the model’s performance.

**Fig. 4.**
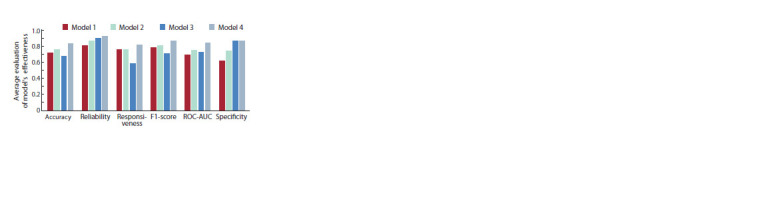
Results of testing four different neural network models on the independent sample.

The performance metrics for different models on the independent test set are shown in Figure 4. According to the test results, structure 4 showed the best results for most selected parameters. Structure 2 also achieved good results. This structure exhibited the lowest sensitivity to overfitting, indicating its higher reliability compared to structure 4.

## Conclusion

In our study, a neural network was successfully developed that classifies individuals into groups practicing or not practicing meditation based on the analysis of their EEG data with an accuracy of approximately 80–85 %. We used an EEG dataset collected and collated during our own experiments, selecting the amplitude of the ERP peak before button press at 250–350 ms and the amplitude value of the peak after button press at 550–900 ms for 64 recording channels. The sample size was 1×2×64.

Four architectures of non-deep convolutional networks were developed, among which structures 2 and 4 performed best in tests on independent data samples. Structure 2, which used a one-dimensional convolutional layer, pooling layer, and a two-layer fully connected network, showed the highest reliability. During the development of this model, it was noted that it was often prone to overfitting due to the limitation of the dataset size. This was mitigated by modifying the structure and scale of the model, specific network initialization parameters, regularization, random deactivation (dropout), and hyperparameter screening of cross-validation.

Overall, the approach proposed by us was tested on two relatively small samples of non-clinical subjects. A similar method on experimental data from the stop-signal paradigm had been previously tested by us in classifying samples of clinical patients with depressive disorders and healthy individuals (Zelenskih et al., 2022). The results of the research presented in this article complement the previous work, as they demonstrate that despite the small sample sizes, the convolutional neural network method allows to achieve a high level of accuracy in classifying different independent groups of people differing in stress levels. Taken together, the results of both studies show that applying neural networks to data obtained from individuals during the stop-signal paradigm is a promising method for assessing their stress levels and the severity of anxiety-depressive symptoms. It should be noted that the results of M.O. Zelenskih and colleagues’ study are based solely on the application of behavioral data obtained in the stop-signal paradigm. The results of our new publication are based on the analysis of brain electrical responses obtained in the same experiment. The continuation of our research should involve the application of convolutional neural networks for the simultaneous analysis of behavioral and neurobiological data in order to more accurately classify participants based on their stress levels.

It is important to note that most standard methods for assessing stress levels or predisposition to anxiety-depressive disorders are based on the use of psychological questionnaires or interviews with a psychiatrist (e. g., Beck et al., 1988). However, such methods have a disadvantage: patients may not want to inform the interviewer about their condition or may inaccurately assess themselves. Inaccurate self-assessment by the patient is often the cause of incorrect conclusions regarding their susceptibility to illness (Nock et al., 2010). Another approach is based on the analysis of behavioral or neurophysiological reactions to emotional stimuli. Such stimuli can be either photographs of faces expressing the patient’s or other people’s emotional states (Quevedo et al., 2016), or emotional messages (Bocharov et al., 2020). This method allows for an objective assessment of the degree of impairment of the brain’s affective functions but is less sensitive to changes in a person’s overall ability to self-control behavior. Our proposed method, on the other hand, is based on the use of non-emotional stimuli to induce a complex sensorimotor reaction that requires either activation or inhibition of movement. Our approach allows for the assessment of the overall level of self-control of behavior but does not provide an opportunity to assess the patient’s affective state. It is obvious that these three approaches (i. e., testing using questionnaires, analysis of reactions to affective stimulation, and analysis of reactions in motor control tasks) are mutually complementary, i. e., they should all be used together for a more detailed assessment of the same patient. Although our proposed approach currently requires further testing, it may yield significant results in the future in the development of diagnostic tools for stress-induced diseases.

## Conflict of interest

The authors declare no conflict of interest.
